# The Role of New 3D Pathology and Lymphocyte Expression of Interstitial Inflammation in Pediatric-Onset Lupus Nephritis

**DOI:** 10.3390/ijms24043512

**Published:** 2023-02-09

**Authors:** Yung-Chieh Huang, Yong-Chen Hsu, Jun-Pen Chen, Lin-Shien Fu

**Affiliations:** 1 Department of Pediatrics, Taichung Veterans General Hospital, Taichung 40705, Taiwan; 2 Department of Pediatrics, National Yang Ming Chiao Tung University, Taipei 11221, Taiwan; 3 Department of Pathology, Taichung Veterans General Hospital, Taichung 40705, Taiwan; 4 Department of Medical Research, Taichung Veterans General Hospital, Taichung 40705, Taiwan; 5 Department of Post-Baccalaureate Medicine, College of Medicine, National Chung Hsing University, Taichung 40227, Taiwan

**Keywords:** pediatric-onset systemic lupus erythematosus (pSLE), lupus nephritis, interstitial inflammation, 3D pathology, Syndecan-1

## Abstract

Lupus nephritis (LN) is a common and severe manifestation of pediatric-onset systemic lupus erythematosus (pSLE). It is one of the major causes of long-term glucocorticoid/immune suppressants use in pSLE. It causes long-term glucocorticoid/immune suppressants use and even end-stage renal disease (ESRD) in pSLE. It is now well known that high chronicity, especially the tubulointerstitial components in the renal biopsy, predicts a poor renal outcome. Interstitial inflammation (II), a component of activity in LN pathology, can be an early predictor for the renal outcome. With the advent of 3D pathology and CD19-targeted CAR-T cell therapy in the 2020s, the present study focuses on detailed pathology and B cell expression in II. We recruited 48 pSLE patients with class III/IV LN to analyze the risk of ESRD based on different II scores. We also studied 3D renal pathology and immunofluorescence (IF) staining of CD3, 19, 20, and 138 in patients with a high II score but low chronicity. Those pSLE LN patients with II scores of 2 or 3 showed a higher risk for ESRD (*p* = 0.003) than those with II scores of 0 or 1. Excluding patients with chronicity >3, high II scores still carried a higher risk for ESRD (*p* = 0.005). Checking the average scores from the renal specimens from different depths, the II, and chronicity showed good consistency between 3D and 2D pathology (interclass correlation coefficient [ICC], II = 0.91, *p* = 0.0015; chronicity = 0.86, *p* = 0.024). However, the sum of tubular atrophy plus interstitial fibrosis showed no good consistency (ICC = 0.79, *p* = 0.071). The selected LN patients with negative CD19/20 IF stains showed scattered CD3 infiltration and a different IF pattern of Syndecan-1 expression. Our study provides unique data in LN, including 3D pathology and different in situ Syndecan-1 patterns in LN patients.

## 1. Introduction

Lupus nephritis (LN) is one of the most common and severe manifestations of systemic lupus erythematosus (SLE), occurring in up to 50% of SLE patients during the course of the disease [1,2,3]. In pediatric-onset SLE (pSLE), the rate of lupus nephritis can be as high as 70% [4]. Lupus nephritis is a major determinant of poor prognosis in pSLE patients due to renal damage itself as well as increased morbidity and costs related to the disease and treatment [5,6].

Renal pathology is a major guide for LN treatment. The World Health Organization (WHO) formalized the LN renal pathology classification in 1974. Even after several times of refinements, the latest 2018 International Society of Nephrology/Renal Pathology Society (ISN/RPS) version maintains most of the original framework [7] and continues to focus on the glomerular alterations to classify LN from I to VI. The addition of the National Institute of Health (NIH) activity and chronicity indices provides a scoring system, but this quantification is still based on glomerular changes [8]. The extent of activity reflects an ongoing injury that may be responsive to therapeutic intervention. Chronicity indicates scarring and irreversible damage that is unlikely to respond to therapy. According to the practice guideline of the International Society of Nephrology/Renal Pathology Society (ISN/RPS) [7], interstitial inflammation (II) is one of the indices of the NIH lupus nephritis activity scoring system. In recent reports, II has been reported to independently predict renal survival [8,9]; however, other reports had inconsistent findings [10,11]. Alsuwaida AO et al. [10] demonstrated no association of II with end stage renal disease (ESRD). Rijnink EC et al. [11] found the components of II, including interstitial infiltrate score, either with lymphocyte or granulocyte dominant, had no impact on ESRD in lupus nephritis. One explanation is the various concurrent chronicity scores noted in these studies. Nevertheless, limited sampling from renal tissue may distort the II score and chronicity; a more detailed study in renal pathology is warranted.

The closely packed T: B aggregates in II were observed in LN patients. Tertiary lymphoid organ formation was noted in about 7% of patients in one series [12]. In addition, further study of the in situ germinal center demonstrated immunoglobulin formation and hypermutation, and the local synthesis antibody was directed to vimentin, rather than the anti-dsDNA antibody for glomerular inflammation [13]. However, more than half of LN patients with interstitial inflammation had T: B aggregates. A recently published study showed that high interstitial B cell densities were associated with the protection of renal survival. In contrast, the high CD4-CD8- T cell densities were associated with acute and chronic renal failure [14]. A post-hoc analysis of the three clinical trials of belimumab (BLISS-SC, BLISS-ENA, and BLISS-76), published in November 2022, showed different kinetics in B/plasma cell subsets after belimumab therapy in LN patients with or without renal flares [15]. These recent publications showed a more complex inflammation in situ in LN.

The present study used the new 3D pathology technique to review its consistency with traditional 2D renal pathology. We also studied the local Syndecan-1 expression in LN patients with different II scores but no significant B lymphocyte infiltration.

## 2. Results

There were 48 pSLE patients with LN classes III or IV. According to their II scores, we divided them into two groups. Those who had a II score of 2 or 3 in their renal biopsy were classified as the high II score group (*n* = 15), and the low II score group included those with a II score of 0 or 1 (*n* = 33). Table 1 shows their demographic profile, laboratory and pathological data, and their renal outcome. The high II score group had higher activity (*p* = 0.00003) and chronicity (*p* = 0.003) than the lower II score group. However, considering the tubulointerstitial components of chronicity—tubular atrophy and interstitial fibrosis—these two groups showed no difference (*p* = 0.119). The group with a high II score had a higher rate of end-stage renal disease (ESRD) (40% vs. 9.1%, *p* = 0.011). If we exclude those with chronicity higher than 3 in both groups, the high II score group had similar chronicity as the low II score group (0.85 ± 1.06 vs. 0.39 ± 0.88, *p* = 0.07), the high II score group still showed a higher ESRD rate (33.3% vs. 3.2%, *p* = 0.005).

Among these 48 pSLE patients with LN, we selected five samples of LN class IV with various II scores for 3D pathology. The control was differently obtained from a patient with a minimal change nephrotic syndrome. Table 2 shows the scores from their original interpretation and the average scores from different depths of the 3D pathology.

Table 3 shows the interclass correlation coefficient (ICC) of different scores in LN between 2D and 3D pathology. Items including endocapillary hyperplasia, cell/fibrocellular crescent, II, and chronicity showed good consistency between 2D and 3D LN pathology. However, the sum of tubular atrophy and interstitial fibrosis, the tubule-interstitial components of chronicity, did not have a good correlation (ICC = 0.79, range from −0.93 to 0.97, *p* = 0.071).

**Figure 1:**
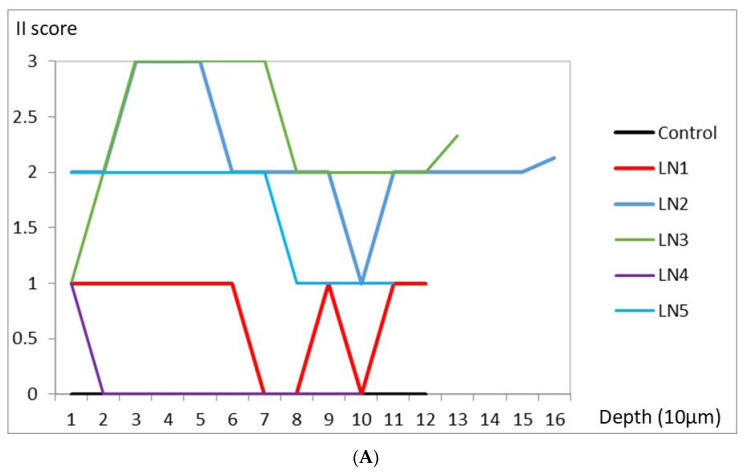

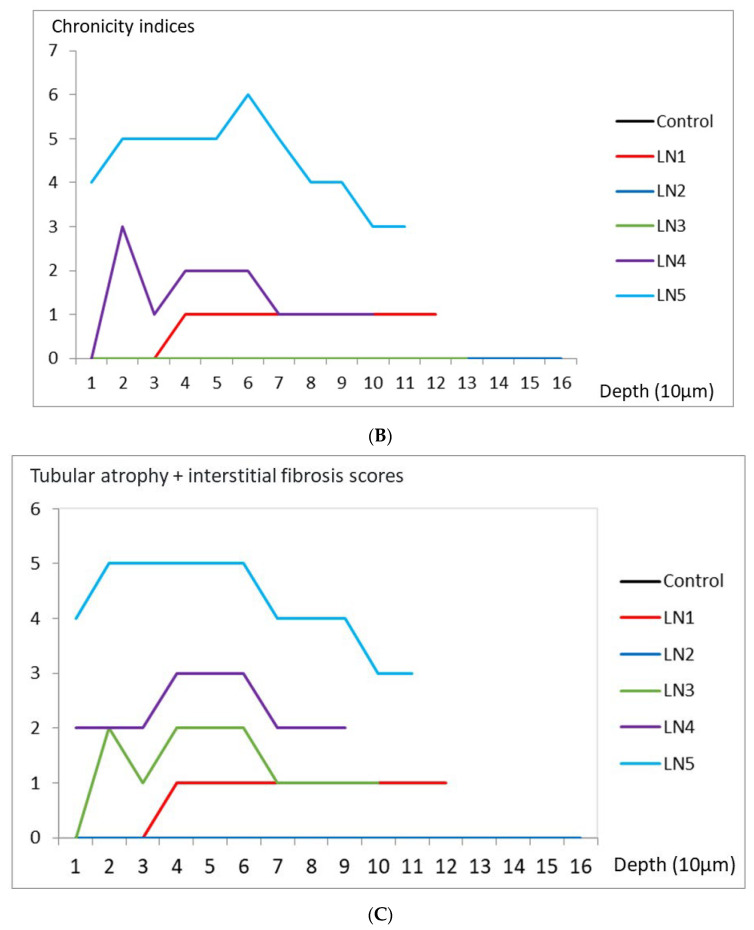
(**A**) II scores from different depths of the renal specimen. (**B**) Chronicity indices from different depths of the renal specimen. (**C**) The sum of tubular atrophy and interstitial fibrosis scores from different depths of the renal specimen.

Figure 1 shows the scores from the different depths of the 3D renal specimen. The II scores are shown in Figure 1A. The chronicity indices were shown in Figure 1B. The sum of tubular atrophy and interstitial fibrosis scores is shown in Figure 1C.

From the analyses above, we can find good consistency in those scores of 0, but some variability did happen in the high scores in LN.

We selected 3 renal specimens lacking CD19/20 expression for Syndecan-1 IF stains. Their clinical and pathological features were summarized in Table 4.

**Figure 2:**
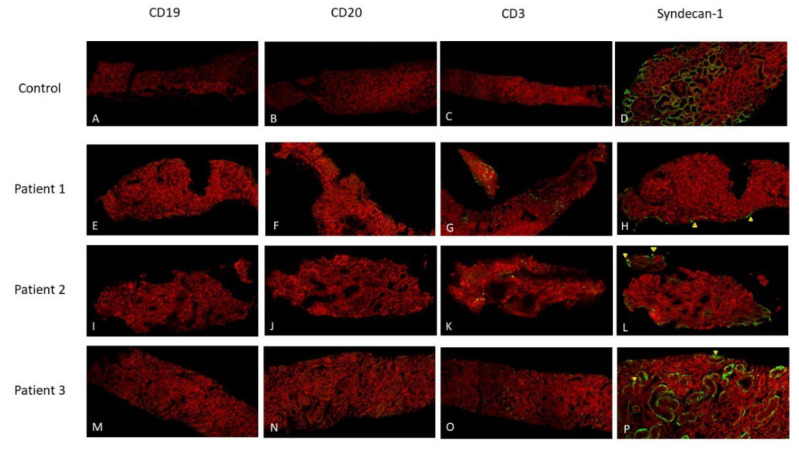
Immunofluorescence stains of CD19 (**A**,**E**,**I**,**M**), CD20 (**B**,**F**,**J**,**N**), CD3 (**C**,**G**,**K**,**O**) and Syndecan-1 (**D**,**H**,**L**,**P**).The positive stain showed in green color. The Syndecan-1 positive cells in interstitial space were marked as yellowish triangles. The scale bar in each subfigure indicates 100 μm.

All 3 LN patients lacked CD19/CD20 expression and had scattered positive CD3 cells in the interstitium. The Syndecan-1 stain in control revealed tubular epithelia. The tubular epithelia expressed much less Syndecan-1 in these 3 lupus nephritis patients. The distributions were uneven, and small amounts of Syndecan-1-positive cells were in the interstitial space, as indicated by the yellowish triangles marked in Figure 2H,L,P.

## 3. Discussion

The present study has provided new images of lupus nephritis that were not previously published. For the first time, we demonstrated that the evaluation of II and chronicity in 3D was applicable to FFPE tissue. This unique tissue-clearing technology is the foundation of this approach. Renal tissue is made optically transparent by immersion in a solution with the same refractive index. This technology requires no use of detergents such as digestive enzymes, sodium dodecyl sulfate, or structure-supporting polymers. Thus, the procedure preserves the native macromolecules in the specimen [16]. Overall, the interclass correlation coefficient was fair between 2D and 3D pathology. Nevertheless, we showed significant variation in scores of II, with chronicity expressed at different tissue depth levels except where the score was zero. In addition to more slices from different depths of the renal specimen, the whole 3D structure, as shown in Supplementary Material Video S1, can provide more data, including the spatial relationship of different interstitial cells for LN study. Now, analysis of the 3D structure with the aid of artificial intelligence in LN is emerging [14].

In a molecular signature study for antibody-secreting cells (ASC) in lupus nephritis published in 2021, a Syndecan-1 positive cell in the renal interstitium was defined as an ASC [17]. There were several studies involving Syndecan-1 in lupus nephritis. Most studies checked the availability of serum Syndecan-1 levels as a marker for lupus nephritis [18,19]. Kim KJ et al. found that serum SDC-1 levels are increased in SLE patients with nephritis [18]. Yu KYC et al. revealed that Syndecan-1 level correlated with the severity of interstitial inflammation [19]. However, from our study, the in situ Syndecan-1 did not show a correlation.

Syndecan-1, also known as CD138, is a marker for plasma cells. It has long been assumed that CD19+CD138+ plasma cell secretes autoantibodies. Mujtahedi SS et al. just published a study in December, 2022, that showed there are several types of long-lived plasma cells contributing to autoimmunity [20], including CD19+/CD138+/CD38Hi (64.1%), CD19−/CD138+/CD38Hi (20.9%), CD19+/CD138−/CD38Hi(10.7%), and CD19−/CD138−/CD38Hi (4.3%), by different days of plasma cell culture. It is not clear if these plasma cells appear in the renal specimens of LN patients. Our study showed a small amount of Syndecan-1-positive cells in a CD19-negative milieu. The presence of CD38 is worthy of further study.

Belimumab, a recombinant human IgG1λ monoclonal antibody, inhibits the B-lymphocyte stimulator. It has been approved for patients with active autoantibody-positive SLE [21,22]. In a post-hoc analysis of three clinical trials of belimumab in SLE, the results showed flared patients who received standard therapy, and displayed less prominent early decreases in CD19^+^CD20^−^CD138^+^ long-lived plasma cells (−11.3% versus −29.2%; *p* = 0.019). Patients with severe flares had less prominent early decreases in CD19^+^CD20^−^CD138^+^ long-lived plasma cells (−23.5% versus −39.4%; *p* = 0.028) and CD19^+^CD27^bright^CD38^bright^ SLE-associated plasma cells (−19.0% versus −27.8%; *p* = 0.045) [15]. From the latest study involving B long-lived plasma cell changes, the results from implied that plasma cells other than CD19+CD138+ play a role in LN.

Treatment of LN has made incremental progress in the past few decades. Despite the tubuloinsterstitial process being an important prognostic factor, patients with lupus nephritis continue to be stratified based on the glomerulocentric ISN/RPS classification criteria to determine therapeutic strategies [23,24]. In the past decade, mycophenolate mofetil (MMF) did improve the rate of complete/partial remission from heavy proteinuria/nephrotic syndrome. However, the response rate is still only 50% [23]. There are still many unmet needs in the treatment of LN, especially in terms of TII. Regular and relatively long-term use of belimumab was demonstrated to decrease flare-ups in LN [24]. In the refractory LN, two cases were reported to be successfully treated after weekly anti-CD38 monoclonal antibody daratumumab for 4 weeks [25]. Daratumumab can kill plasma cells and modulate effector T cell responses. Furthermore, the recent success of anti-CD19 chimeric antigen receptor (CAR) T cell therapy in five refractory SLE patients was just reported in October 2022. All these 5 SLE patients had LN classes III or IV, and proteinuria was significantly improved after cell therapy for 5–17 months [26]. With the advent of detailed studies about B plasma cell markers, we can choose candidates for different therapies more precisely.

There are several limitations to this study. The number of 3D pathologies is limited. The technique has never been applied to human renal tissue. We only checked limited representative specimens. The selection criteria included the class IV LN in the ISN/RPS classification, various scores of II and chronicity, and the adequate thickness of the renal sample. This study only provides comparative data between traditional 2D pathology and this de novo 3D pathology. We used the “pseudo-needle” track to check the depth adequate for immunofluorescence (IF) staining of interstitial lymphocyte markers. The 3D IF stains for Syndecan-1 or CD38 are not available at present.

## 4. Materials and Methods

### 4.1. Patients

From January 2006 to December 2016, we recruited 48 patients who fulfilled the American College of Rheumatology classification criteria for the diagnosis of SLE [27] before the age of 18 and had the diagnosis of LN class III or IV confirmed by renal biopsy in our hospital.

All of the 48 patients received glucocorticoid pulse therapy, with one or more than one immunosuppressants (IS). The IS included cyclophosphamide, mycophenolate mofetil (MMF), and cyclosporine. We evaluated their renal conditions, including proteinuria and long-term renal outcome, using the criteria from the KDIGO practice guideline for glomerulonephritis [24].

This study was approved by the Institutional Review Board of Taichung Veterans General Hospital (IRB: SE22117A).

### 4.2. 3D Pathology

**Figure 3:**
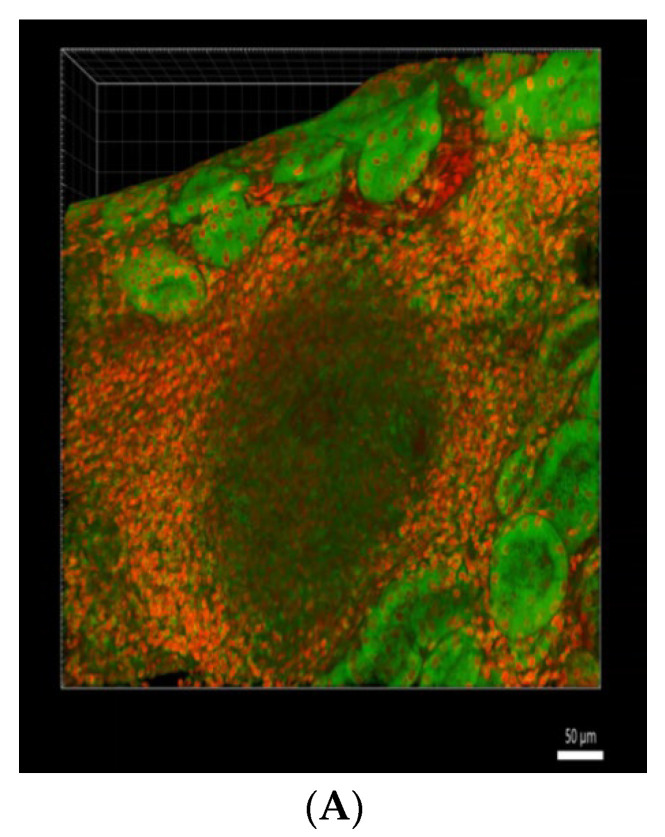

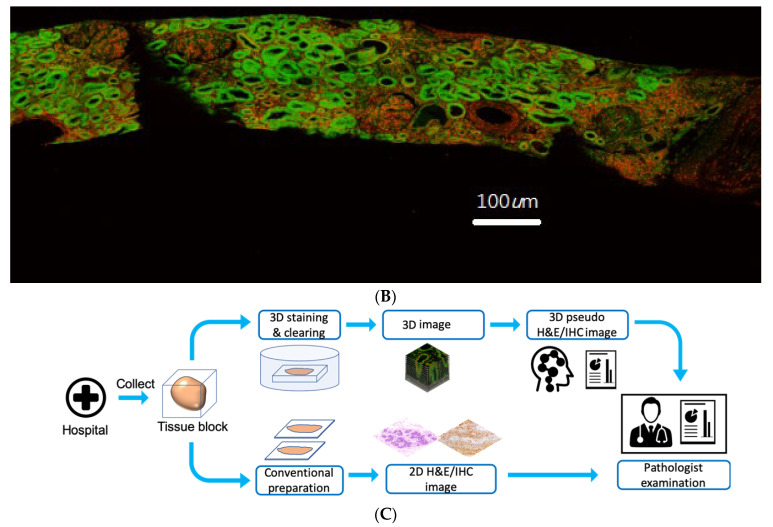
(**A**) 3D image for cell nuclear and membrane of renal tissue. (Cell nuclei were stained as red and membrane as green.). (**B**) Cropped 2D image after “pseudo-needle” track procedure from 3D tissue image. (**C**) Workflow of tissue processing, staining, and final output of the 3D image results.

Sections of 100 to 150 μm thickness were made from formalin-fix paraffin-embedded (FFPE) renal tissue specimens, de-waxed and rehydrated, followed by an overnight treatment with Triton X-100 (2%), lipophilic tracer DiD (20 μg/mL, cat, D307, ThermoFisher Scientific, Waltham, MA, USA) for cell membrane staining for 8 h, and the mixture containing a proprietary clearing solution (JelloX Biotech, Zhubei, Taiwan) and 4′,6-Diamidino-2-phenylindole dihydrochloride (DAPI) (5 μg/mL, cat, D9542, Sigma-Aldrich, Burlington, MA, USA) at RT overnight for optical transparency and nuclear staining. Subsequently, the sample was carefully transferred to cambered coverslips for 3D image acquisition using confocal microscopy. Each image was measured by CLSM Z-stack scanning (pinhole: 235 μm; Z interval: 0.7 μm between consecutive layers). The 3D image from the above processes is shown in Figure 3A. The panoramic view of the 3D block is demonstrated in Supplementary Material Video S1.

### 4.3. “Pseudo-Needle Biopsy” from 3D Pathology Specimen

For the renal biopsy specimens, the 3D image of the 110 to 150 μm- thick processed tissue was obtained as described in the above paragraph. We chose to create a “pseudo-needle biopsy” from this image to simulate the application of the technology in needle biopsy specimens. The “pseudo-needle” track was designed to be 8 mm × 1 mm and cover the entire 150 μm in the *z*-axis. We used the ImageJ software (version 1.53a, National Institute of Health, Bethesda, MD, USA) to create the pseudo-needle biopsy images every 1μm from the top to the bottom of the 3D specimen. The cropped images (Figure 3B) were then exported using Imaris 9.7 software (Oxford Instruments, Abingdon, UK). The exported images in Figure 3C summarized the procedures of 3D image acquisition and pseudo-needle renal biopsy.

### 4.4. Interpretations of Renal Pathology

The histological characteristics of each renal biopsy were interpreted according to the ISN/RPS classification [28]. We divided patients into two groups according to their interstitial inflammation scores (II score)—0, 1 as a low II score; and 2, 3 as a high II score. Their activity and chronicity were compared.

The pathologist (Shu YJ) also checked cropped 2D nuclear/membrane images every 10μm by the ISN/RPS Classification. The checked items contained all components of chronicity and parts of activity, including II, endocapillary hyperplasia, and cellular/fibrocellular crescent. The fibrinoid necrosis, hyaline deposit, and neutrophils/karyorrhexis cannot be interpreted clearly in the cropped 2D nuclear/membrane images.

### 4.5. Immunofluorescence Stain

Sections of 4 μm thickness were made from formalin-fix paraffin embedded (FFPE) of a selected renal biopsy specimen, de-waxed, antigen retrieved, and subjected to blocking nonspecific antibody binding by immersion in a reagent containing PBS, 3% BSA, 0.5% Triton X-100, and 0.02% NaN_3_ for 1 h. The section was separately treated with primary monoclonal antibodies specific for CD3 (clone SP162, 1:150, Abcam, Cambridge, UK), CD19 (clone C1C3, 1:250, GeneTex, Irvine, USA), CD20 (clone L26, 1:50, Abcam, Cambridge, UK), and Syndecan-1 (clone SP152, 1:50, Abcam, Cambridge, UK) at 25 °C for 1 h and washed with 1% Triton X-100 in PBS for 30 min. Bound primary antibodies were detected via poly-HRP-conjugated goat anti-rabbit/mouse IgG (ready-to-use, 50–100 μL, ThermoFisher Scientific, Waltham, MA, USA) at room temperature (RT) for 1 h. Nuclei staining was carried out by treatment with DAPI (5 μg/mL, cat, D9542, Sigma-Aldrich) at RT for 30 min. Each sample was carefully transferred to cambered cover slips for 2D image acquisition using an FV3000 confocal laser scanning microscope (Olympus, Tokyo, Japan) with FV31S-SW software (Olympus, Tokyo, Japan).

### 4.6. Statistics

Statistical analysis was completed using the chi-square test and Fisher’s exact test to check for any difference in percentages under various conditions. The Mann–Whitney U test was used to compare those continuous variables between the two groups. A *p* value less than 0.05 was considered to be significant. We calculated the interclass correlation coefficient to check the consistency between 2D and 3D pathology scores. (SPSS Version 22; SPSS Inc., Chicago, IL, USA).

## 5. Conclusions

The present study showed the usefulness of 3D pathology in studying LN, especially interstitial inflammation, which is the key to chronic change and end-stage renal diseases. Aside from T:B lymphocyte aggregates or tertiary lymphoid organ formation in the renal interstitium, the in situ Syndecan-1 expression and related CD19−CD138+ and CD19−CD138− studies in LN merit further investigation.

## Figures and Tables

**Table 1:** Basic profile, serological, laboratory and pathology data in low and high interstitial inflammation scores groups.

	Low Interstitial Inflammation Score (0, 1) (*n* = 33)	High Interstitial Inflammation Score (2, 3) (*n* = 15)	*p* Value
Age of diagnosis, years	14.41 ± 2.46	14.33 ± 2.71	0.512
Gender (Male:Female)	11:22	1:14	0.039
Age of renal biopsy	18.23 ± 4.15	17.48 ± 2.56	0.317
Follow-up period, months	81.4 (37.1–114.9)	92.8 (41.3–154.6)	0.074
**Data at renal biopsy** Positive Anti-dsDNA antibody	27 (81.9%)	10 (66.7%)	0.1667
C3, mg/dL	68.6 ± 30.5	79.1 ± 37.2	0.239
C4, mg/dL	6.2 ± 6.8	7.6 ± 7.1	0.081
Leukopenia	8 (24.4%)	4 (26.7%)	0.8753
Thrombocytopenia	6 (18.2%)	1 (6.7%)	0.2948
Urine protein/creatinine	3.77 ± 4.34	4.47 ± 2.98	0.314
Serum creatinine	1.07 ± 0.53	1.71 ± 0.87	0.0033
**Renal pathology**			
End stage renal disease	3(9.1%)	7 (46.7%)	0.003
Activity	5.5 ± 3.59	11.5 ± 5.35	0.00003
Chronicity	1.45 ± 0.59	1.67 ± 2.38	0.003
Tubular atrophy + Interstitial fibrosis	0.48 ± 1.09	0.93 ± 1.44	0.119
Class IV	18 (54.5%)	12(80%)	0.089
Class IV + V	4(12.2%)	3 (20%)	
Class III	9 (27.3%)	0	
Class III/V	2 (6.1%)	0	

ANA: antinuclear antibody; Anti-dsDNA antibody: anti-double-stranded DNA antibody; C3: complement component 3; C4: complement component 4.

**Table 2:** Comparison of 2D and 3D average scores of interstitial inflammation, chronicity, and items in chronicity in control and pSLE patients with lupus nephritis class IV.

Patient	Interstitial Inflammation	Chronicity	Tubular Atrophy	Interstitial Fibrosis	Global/Segmental Sclerosis	Fibrous Crescent
Control	0/0 *	0/0	0/0	0/0	0/0	0/0
1	0/0.75	2/0.75	1/0.75	1/0	0/0	0/0
2	0/0.1	3/1.4	1/0.9	1/0.4	1/0.1	0/0
3	1/1.64	3/4.45	1/2.45	1/1.82	1/0.18	0/0
4	3/2.133	0/0	0/0	0/0	0/0	0/0
5	3/2.333	0/0	0/0	0/0	0/0	0/0

* Scores from original pathology interpretation/ average scores from different depth in 3D pathology.

**Table 3:** Interclass correlation coefficient of different lupus nephritis pathology scores.

	ICC	95%CI		*p* Value
		Lower	Upper	
EH	0.96	(0.72–	0.99)	0.002 **
C/FC crescents	0.99	(0.92–	1.00)	<0.001 **
II	0.91	(0.28–	0.99)	0.015 *
Chronicity	0.86	(0.17–	0.98)	0.024 *
TA + IF	0.79	(−0.93–	0.97)	0.071

ICC: Interclass correlation coefficient. EH: endocapillary hyperplasia. C/FC crescents: cellular/ fibrocellular crescents. II: interstitial inflammation. TA + IF: tubular atrophy+ interstitial fibrosis. * *p* < 0.05; ** *p* < 0.01.

**Table 4:** Data of the three lupus nephritis patients for the Syndecan-1 stain.

	Class	II	Chronicity	TA + IF	Activity	Cr(mg/dL) *	Anti-ds DNA	Renal Outcome
Patient 1	III + V	0	2	1	1	0.7	+	Normal Cr UPCr > 1 ^#^
Patient 2	IVG + V	3	0	0	15	2.08	+	ESRD ^%^
Patient 3	IVG	1	3	2	5	2.03	+	ESRD

* Cr: serum creatinine level at renal biopsy. ^#^ UPCR: urine protein/creatinine ^%^ ESRD: end stage renal disease.

## Data Availability

The data presented in this study are available on request from the corresponding author.

## Supplementary Materials

The following supporting information can be downloaded at: https://www.mdpi.com/article/10.3390/ijms24043512/s1.
